# Management algorithm of external fixation in lower leg arterial injury for limb salvages

**DOI:** 10.1186/s12893-022-01486-2

**Published:** 2022-03-03

**Authors:** Lei Jin, Song Zhang, Yuxuan Zhang, Xin Lin, Dehong Feng, Kejia Hu

**Affiliations:** 1 Department of Orthopedics, Wuxi Hongqiao Hospital, Jiangnan University School of Medicine, Wuxi, 214026 China; 2 Department of Orthopedics, Wuxi Orthopedics Hospital, Soochow University, Wuxi, 214062 China; 3 School of Basic Medicine, Naval Medical University, Shanghai, 200433 China; 4 Department of Orthopedics, Wuxi People’s Hospital, Nanjing Medical University, Wuxi, 214023 China; 5 Laboratory of Digital Medicine, Wuxi People’s Hospital, Nanjing Medical University, Wuxi, 214023 China; 6 Ruijin Hospital, Shanghai Jiao Tong University School of Medicine, Shanghai, 200025 China

**Keywords:** External fixation, Lower legs, Arterial injury, Bone nonunion, Flap transfer

## Abstract

**Purpose:**

The aim of this study is to investigate the outcome of these limb-threatening injuries through external fixation treatment and to discuss the case of patients’ functional recovery after external fixation.

**Methods:**

Demographics, surgical treatment and outcomes in 88 patients with lower leg arterial injuries treated by external fixation at two trauma centers from 2009 to 2018 were reviewed. The primary outcome was the rate of successful lower leg salvage, while secondary outcomes were complications and functional recovery.

**Results:**

Eighty-eight patients were identified and 80 patients (90 legs) maintained a successful lower leg salvage. The mean age was 32.7 ± 10.8 years, and 81.8% were male. The primary outcomes included the following complications: pin-tract infection (8 legs), pins loosening (4 pins), wound superficial infection (7 legs), deep infection developed osteomyelitis (3 legs), bone nonunion or bone defect (17 legs) and amputation (8 legs). The average healing time of fracture was 5.6 ± 4.3 months. The maintain of external fixation average time was 5.8 ± 3.6 months. The improvement of scores of the pain, function and quality of life in our follow-up was statistically significant.

**Conclusion:**

For the lower extremity fracture patients with vascular injuries, using external fixation correctly can improve clinical outcomes and produce the improvement of pain, function and the quality of life.

***Level of evidence*:**

Retrospective cohort, level IV.

## Highlights

- The success rate of the lower leg salvage is high, reach the percentage of 91.8% (90/98).
- External fixation is less invasive, with achieving adequate stability to repair the arterial injury timely, can lower the ischemic time, and beneficial for the following bone or soft tissue repair.
- Treating the patients with external fixators timely is beneficial to the following vascular anticoagulation, bone defect and vein graft, as a result, the protection of lower limb can be improved.

## Introduction

Limb loss following lower leg arterial injury is common and has serious implications for the patient’s life and functionality. The lower leg arterial injury is sometimes accompanied by comminuted fracture, severe wound contamination, etc. It can easily lead to severe complications such as compartment syndrome, bone exposure infection, amputation, which can lead to damage to patients’ life, and limb salvage is critically dependent on ischemic time [1].

External fixation for lower limb fractures is an essential tool in the armamentarium of the trauma surgeon in acute trauma [2, 3]. The main indication is to control damage through temporary fracture stabilization. The goal is to safeguard and reconstruct the alignment, length, and rotation of the fractured limb [2]. The use of external fixation is less invasive, can achieve adequate stability, and provide good access for wound management without compromising stability [4].

Previous studies indicated that limb loss after lower leg arterial injury was fatal if left untreated or treated untimely. The use of external fixation can yield excellent stability to allow the vascular repair to be performed in a controlled environment to protect the completed vascular repair from disruption [5]. Nevertheless, the current research paid more attention to the complex lower extremity deformity correction [6–9], the modification for external fixation [10–12] or case report of the use of external fixators in fractures [11]. However, according to our knowledge, to date, there has been no published work regarding the outcome of the treatment of external fixators in lower extremity arterial injuries. In other words, this is quite difficult to access the effects of external fixators used in treating lower limb arterial injuries.

The aim of this study is to investigate the limb salvage outcome, functional results of these limb-threatening injuries through external fixation treatment, and to discuss the case of patients’ functional recovery after external fixation.

## Materials and methods

From January 2010 to December 2018, trauma patients with lower leg arterial injury which surgically treated with external fixation by the Microsurgery team in two level 1 trauma centers (Wuxi People’s Hospital and Wuxi Orthopedic Hospital) were retrospectively included. This study was performed with institutional review board approval in accordance with the Helsinki Doctrine.

Two separate reviewers performed the data collection, recording patient demographics, medical comorbidities, injury mechanism, Gustilo–Anderson classification [13], mangled extremity severity score (MESS) [14], injury severity score (ISS) [15], AIS abbreviated injury scale [16], time to surgery, flap use in soft-tissue reconstruction, follow-up time, and postoperative complication. The primary outcome of interest was lower leg salvage. Secondary outcomes included complications and functional recovery. And we use the Lower Extremity Functional Scale (LEFS) to evaluate the functional recovery of lower limbs, the Visual Analogue Scale (VAS) and the Quality of Life Scale (QOL) to evaluate pain and life quality correspondingly.

Statistical analyses were performed using Stata version 14.0 MP (StataCorp) to assess for differences in patient demographics, injury characteristics, treatment course, and complications. The Kolmogorov-Smirnoff test was used to test whether the data were normally distributed. Normally distributed data were expressed as a mean ± standard deviation, and skewed data were expressed as median (interquartile range). F-test was used for homogeneity of variance, independent samples t-test for equal variance, and the non-parametric test was used for unequal variance. P < 0.05 was considered statistically significant.

## Results

### Patients demographics

Eighty-eight patients (98 legs) were diagnosed with a lower leg arterial injury with an unstable bone fracture or knee dislocation, which surgically treated with external fixation was included in the study. Table 1 presents the demographic data for the study population: age, gender, etc.

**Table 1:** Demographics of patients with lower leg arterial injury

Demographics	Case (N = 88)
Age, years, mean ± SD	32.7 ± 10.8, (range 16–65)
Gender	
Males	72
Females	16
The mechanisms of injury	
Motor vehicle accident trauma	40
Bruise injury caused by heavy objects	34
Falling injury	3
Twist injury by working machines	8
Cutting injury	1
Explosion injury	2
The types of arterial injury	
Popliteal artery	35 (36 legs)
Anterior tibial artery	16 (20 legs)
Posterior tibial artery	17 (20 legs)
Anterior and posterior tibial artery	20 (22 legs)
The types of skeletal fracture	
Upper 1/3 tib/fib	40 (44 legs)
Middle 1/3 tib/fib	18 (22 legs)
Distal 1/3 tib/fib	28 (30 legs)
Traumatic knee dislocation	2 (2 legs)
Soft tissue injury	
Open (Gustilo IIIc)	80 (88 legs)
Closed	8 (10 legs)
Injury evaluation	
AIS score, mean ± SD	10.1 ± 1.0, (range 9–12)
MESS score, mean ± SD	5.8 ± 1.4, (range 2–10)
ISS score, mean ± SD	18.5 ± 2.5, (range 15–22)
Injury to Surgery time, hours, mean ± SD	5.5 ± 3.2, (range 3–12)

N number of patients, SD standard deviation, AIS abbreviated injury scale, MESS mangled extremity severity score, ISS injury severity score; Data were reported as the number (injured legs) of patients except where noted

**Fig. 1:**
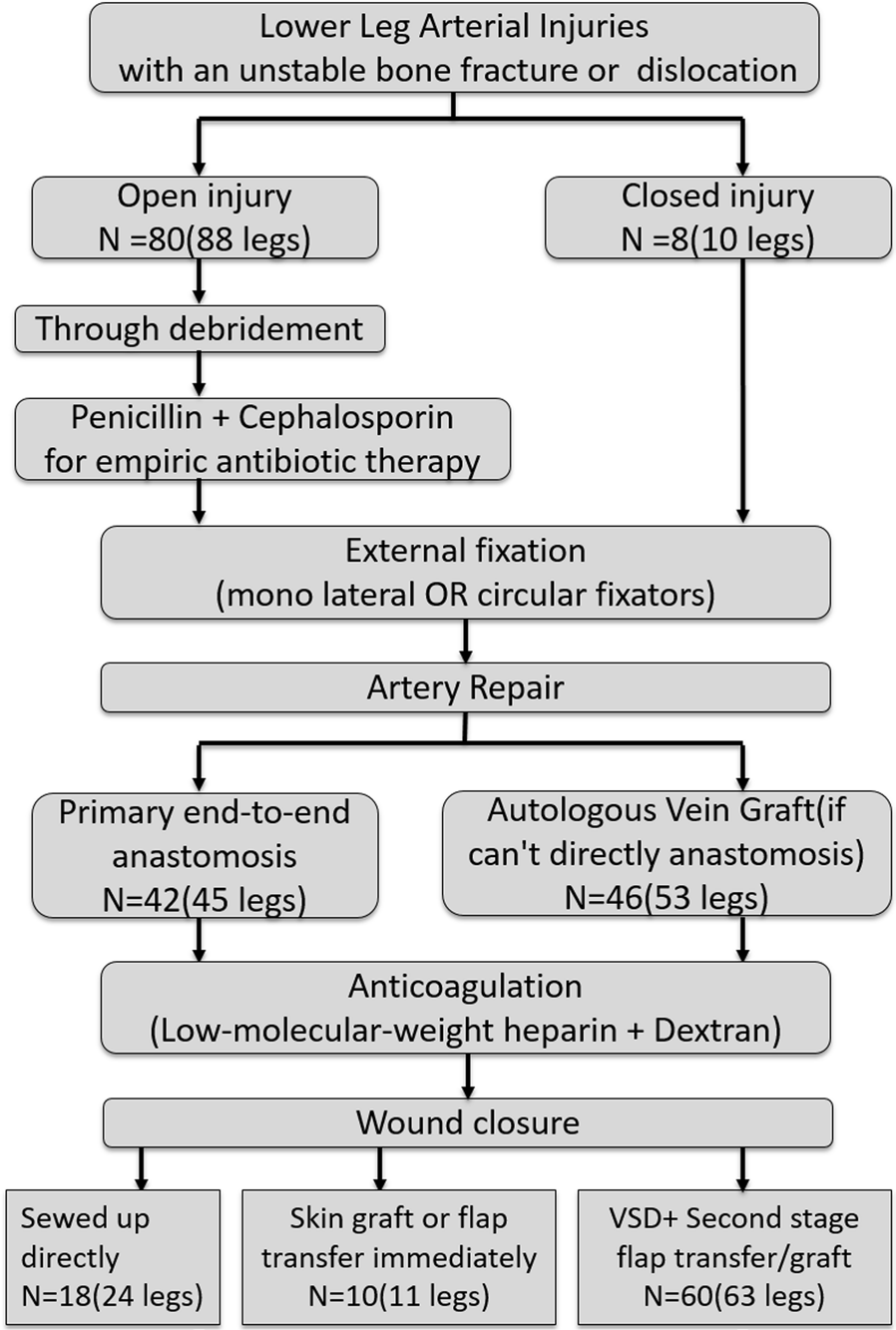
We initially classify the patients and give people who suffer from open injury empiric antibiotic therapy. After that, we give patients external fixation and estimate their prognosis after the anticoagulation and wound closure

Patients who suffered from lower leg atrial injuries with an unstable bone fracture or dislocation were classified into open injury and closed injury. Then, all the patients were treated with external fixation. And our study used Primary end-to-end anastomosis and Autologous Vein Graft to repair the artery. Lastly, we classified the wound closure into different situations (Fig. 1).

### Primary outcomes

**Fig. 2:**
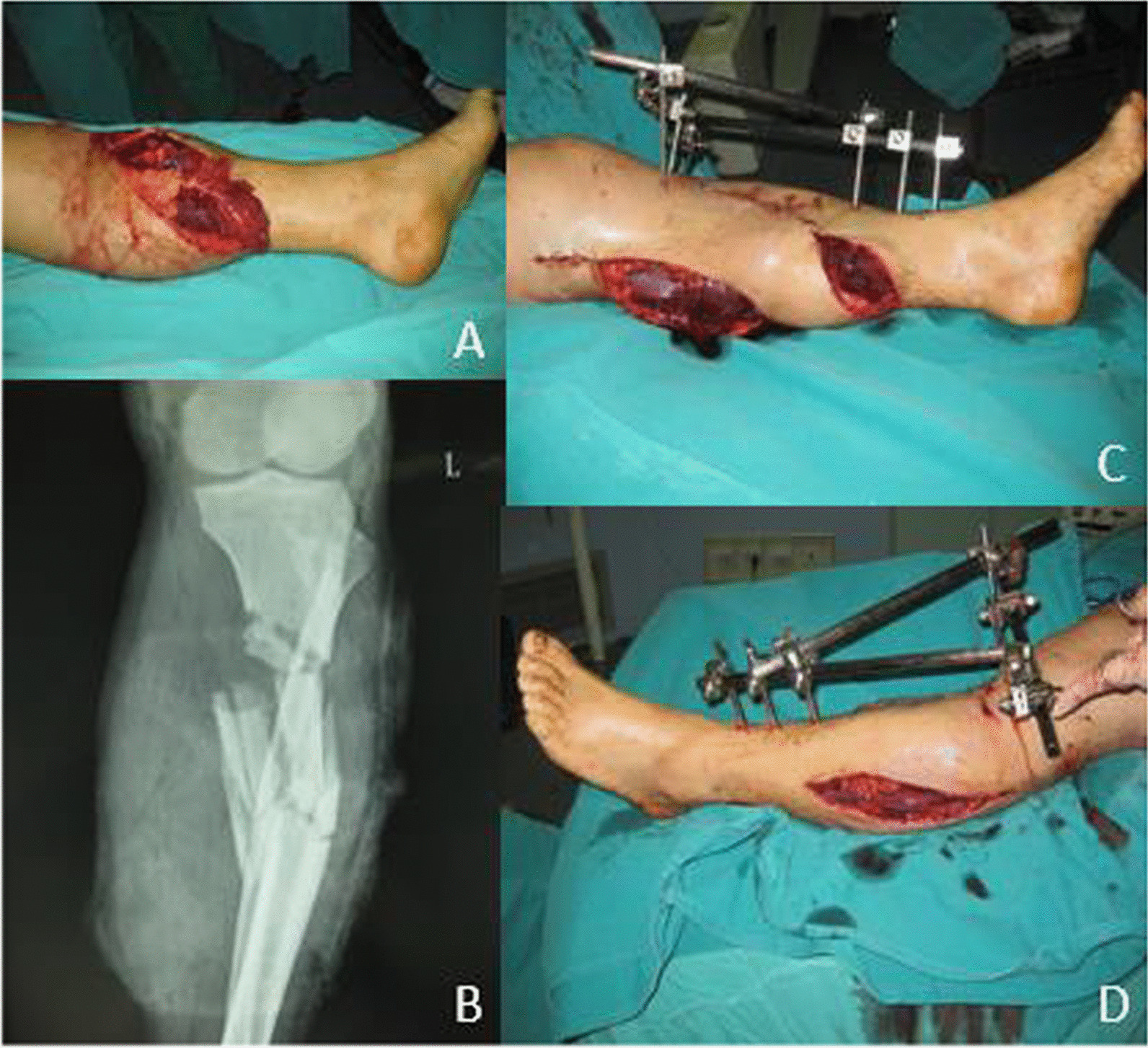
An example of the classification and the application of external fixation. **A** The patient was classified as type IIIc by Gustilo classification. **B** The preoperative radiograph showed an open comminuted fracture of the left tibia and fibula. **C, D** External fixation was used

External fixation (including mono lateral and circular Fixators) was performed in all the 88 patients prior to arteries repair. We divided the patients into different parts by Gustilo classification. An example of the classification was presented (Fig. 2).

**Fig. 3:**
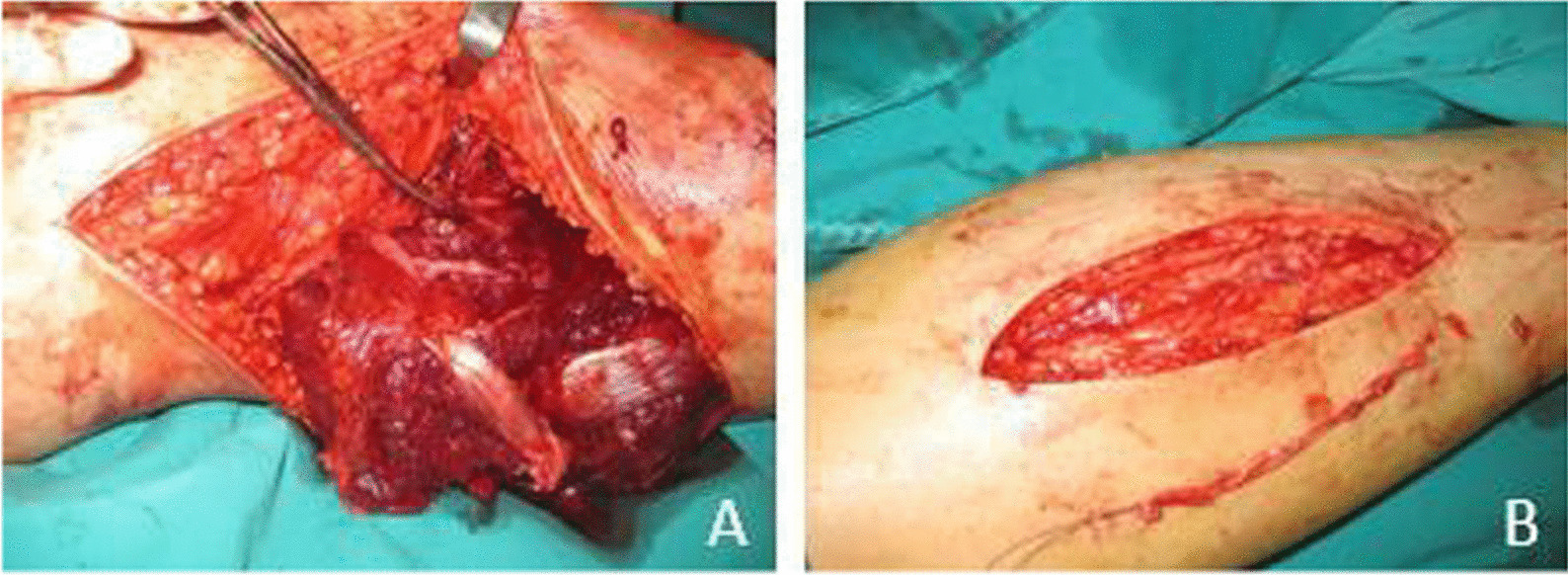
**A** The popliteal artery was completely lacerated and shortened. **B** Autologous vein graft from the contralateral leg was performed

Primary end-to-end anastomosis under the microscope was preferred in 42 patients. Autologous vein graft from the contralateral leg was used in 46 patients due to the artery was shortened after thorough debridement (Fig. 3).

No patients died in this study, 8 patients (8 legs) underwent secondary amputation due to the onset of artery blood-circulation crisis postoperatively, no sign of revascularization after being treated by spasmolytic medicine, or transplantation of the contralateral greater saphenous vein. All the 8 patients were in a poor general condition, who had a MESS score of 8 or above (Table 2).

**Table 2:** Complications of lower leg arterial injury patients treated with external fixation

Complication	N (45 legs)	Treatment
Pin tract infection	6 (8 legs)	Effective antibiotics
Pins loosening	2 (2 legs)	Exchanged the pins
Post-operative wound		
Superficial infection	7 (7 legs)	Effective antibiotics
Deep infection developed osteomyelitis	3 (3 legs)	Thorough debridement, sequestrectomy and bone cement placement with vancomycin
Bone nonunion or bone defect	16 (17 legs)	Bone grafting or bone transporting
Crisis of blood-circulation	8 (8 legs)	Amputation

Eighty patients (90 legs) obtained successful limb salvage, and the salvage rate was 91.8%. 80 patients were followed up for 12 months to 3 years, with an average of 15.5 ± 5.5 months. The external fixation time was 4 to 12 months, with an average of 5.8 ± 3.6 months. The average healing time of fracture was 5.6 ± 4.3 months, ranging from 3 to 13 months.

### Complications

Minor complications included pin-tract infection, pins loosening and wound superficial infection.

Three patients (3 legs) with a deep infection developed osteomyelitis, treatment involved thorough debridement, sequestrectomy and bone cement placement with Vancomycin, when the infection was quiescent as indicated by inflammatory parameters at least 1 year later, removal of External fixation and bone grafting with internal plating fixation was performed. All of them got bone union postoperatively in 7 to 15 months, with an average of 10.6 ± 2.1 months.

**Fig. 4:**
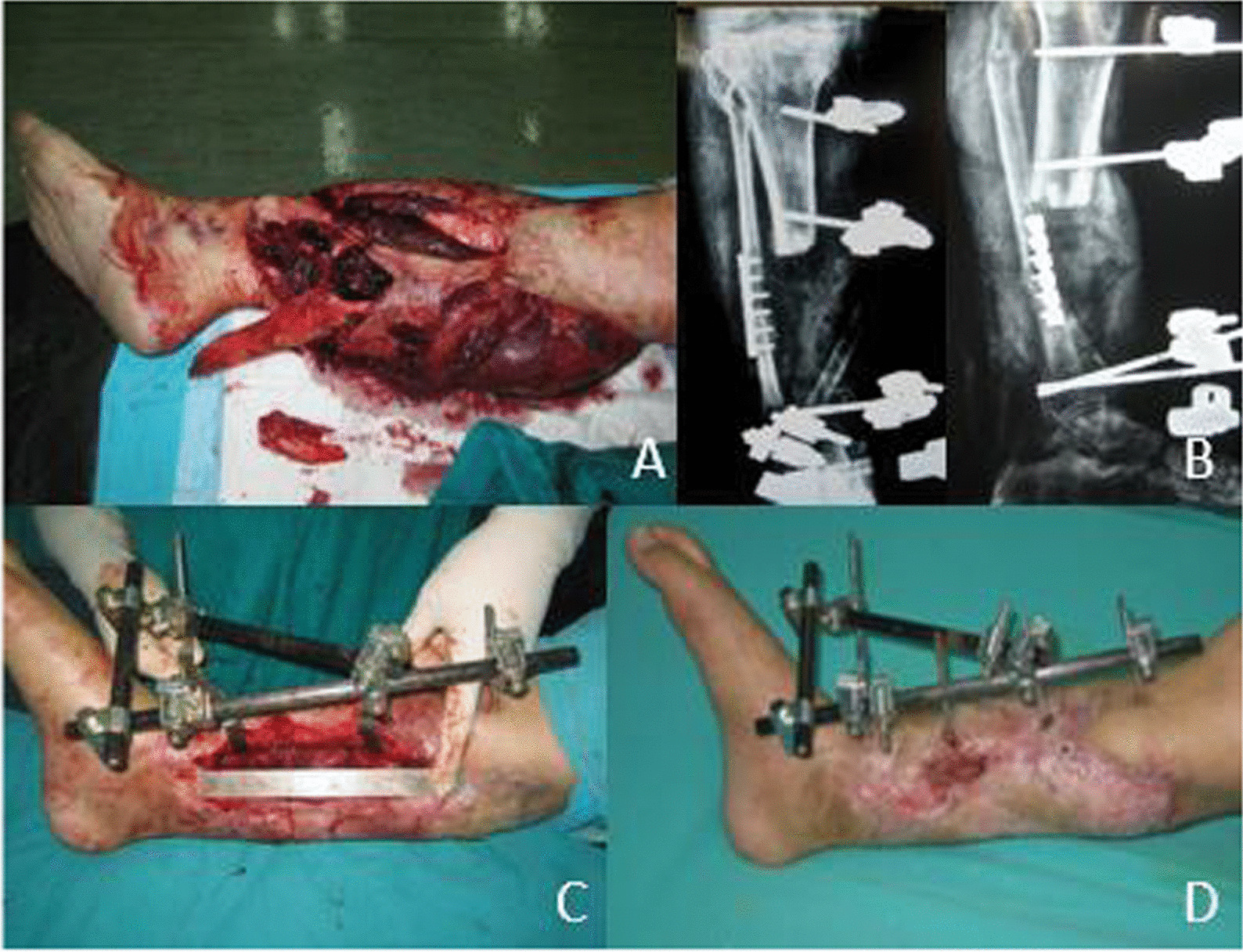
The use of external fixation and the outcome of the patient from Fig. 3. **A** The patient was classified as type IIIc by Gustilo classification. **B** External fixation was used after vascular repair. **C** 12 months postoperative appearance after external fixation. **D** The tibia was shortened about 14 cm

Sixteen patients (17 legs) suffered bone nonunion, 6 to 12 months after wound healing, patients were performed removal of External fixation, internal plating fixation replacing and bone grafting or bone transporting, all of them got bone union postoperatively in 5 to 14 months, with an average of 9.2 ± 3.2 months. Among them, 5 patients (5 legs) who had unilateral limb shortening experienced surgery. The length of the tibial bone defect was 4.5–14.0 cm with an average of 7.2 ± 3.8 cm (Fig. 4).

**Fig. 5:**
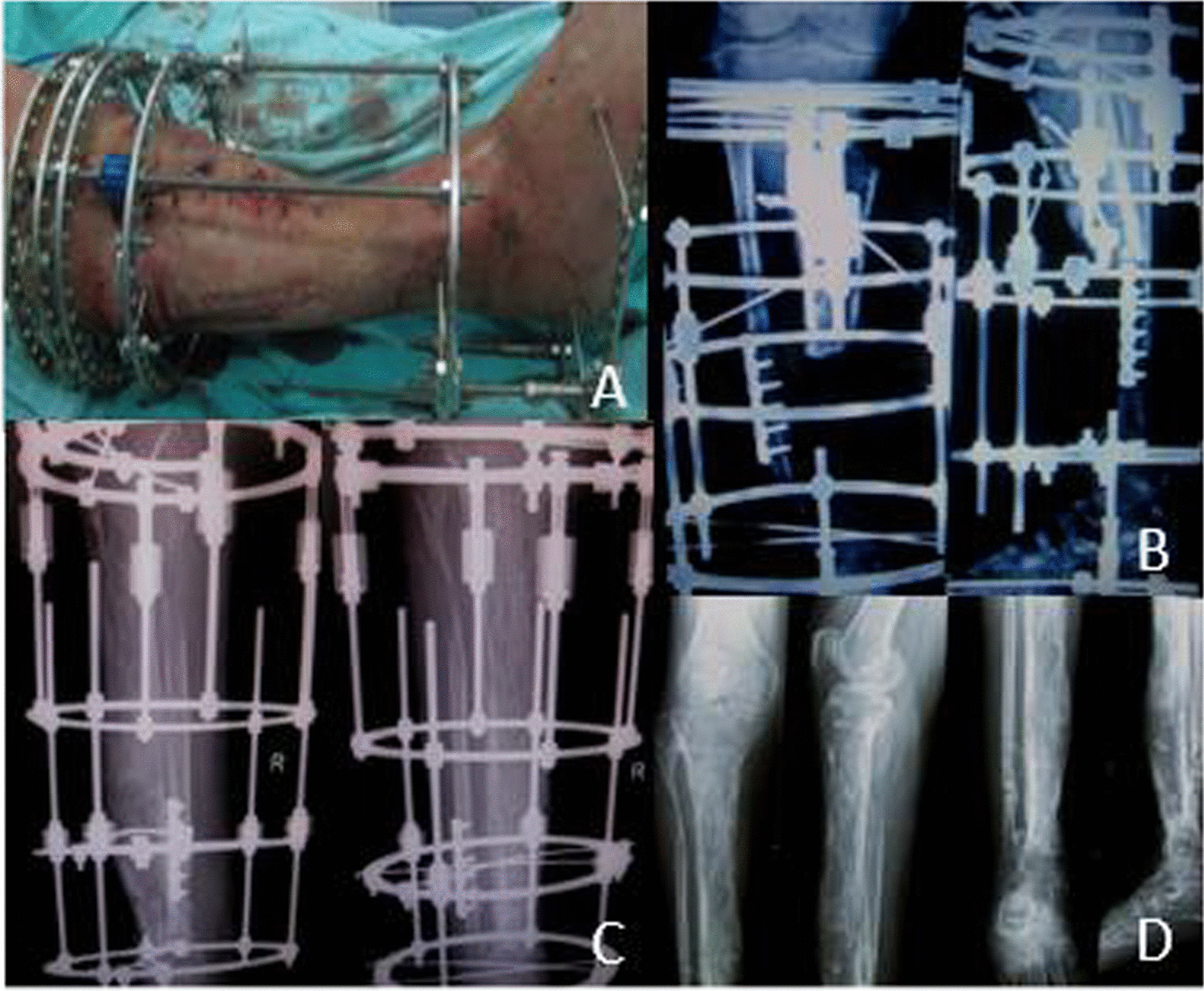
Imaging method was used to evaluate the prognosis of the patients. **A** Most of the wound was closed during shortening-lengthening operation. **B** Postoperative radiograph showed a good external fixation. **C, D** Anteroposterior and lateral X-ray film at 12 months after operation, and after the removal of fixators

Limb shortening-lengthening method using Ilizarov technique (external circular Fixators) was applied. At the time of the latest follow-up, all patients had shortened length less than 2 cm (Fig. 5).

### Functional recovery

**Fig. 6:**
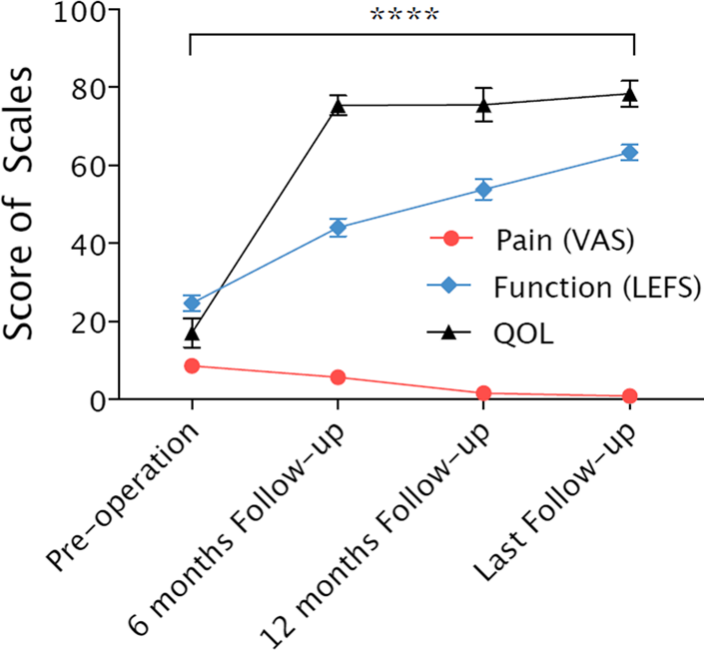
The scale of VAS, LEFS, QOL from the pre-operation to follow-up time. The VAS, LEFS and QOL score of the patient from Fig. 4 were detected and statistical tests were conducted. We can see the tendency of the scores: the VAS score was decreased, whether the LEFS and QOL scores were increased over time. Statistical tests were conducted on the three groups of data, and it had a statistical difference between the group (pre-operation) and the group (last follow-up)

The LEFS was used to evaluate the functional recovery of lower limbs with artery injuries in 80 patients (90 legs), while pain and quality of life were accessed by VAS and QOL scale separately. The survey measurements of the scales were shown below (Fig. 6). All three scales tend to be improved overall three follow-up time points.

**Fig. 7:**
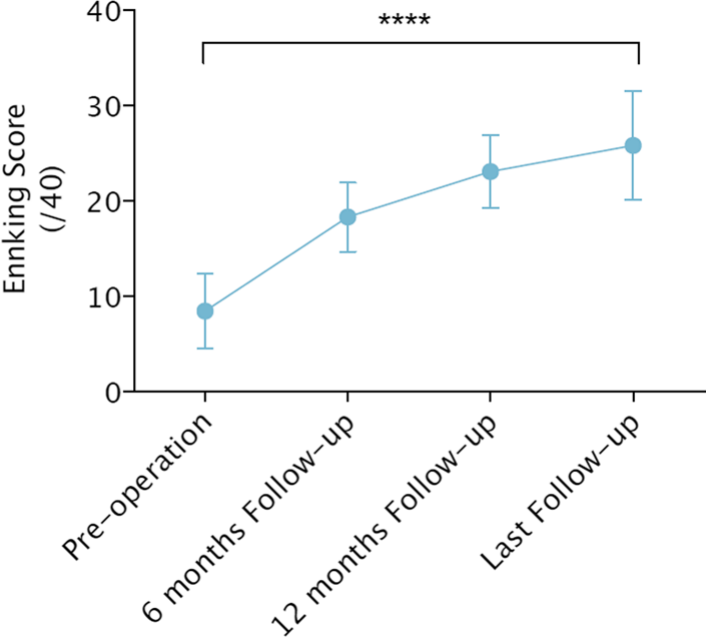
The Enneking score from pre-operation to follow-up time. The Enneking score was increased over time, and different follow-up time’s scores had statistical differences

The functional outcome of 70 patients (78 legs) following reconstruction with free tissue transfer or bony union was evaluated using the Enneking score system [17]. The latter is determined by clinical examination and based on an assessment of the degree of physical disability and psychological acceptance of the reconstruction. The Enneking score is expressed as a percentage of the non-injured contralateral limb and was measured routinely at the orthopedic clinics. The mean Enneking score for patients with vascular injury was 23.8 ± 12.5, with a range of 7 to 38 (Fig. 7).

**Fig. 8:**
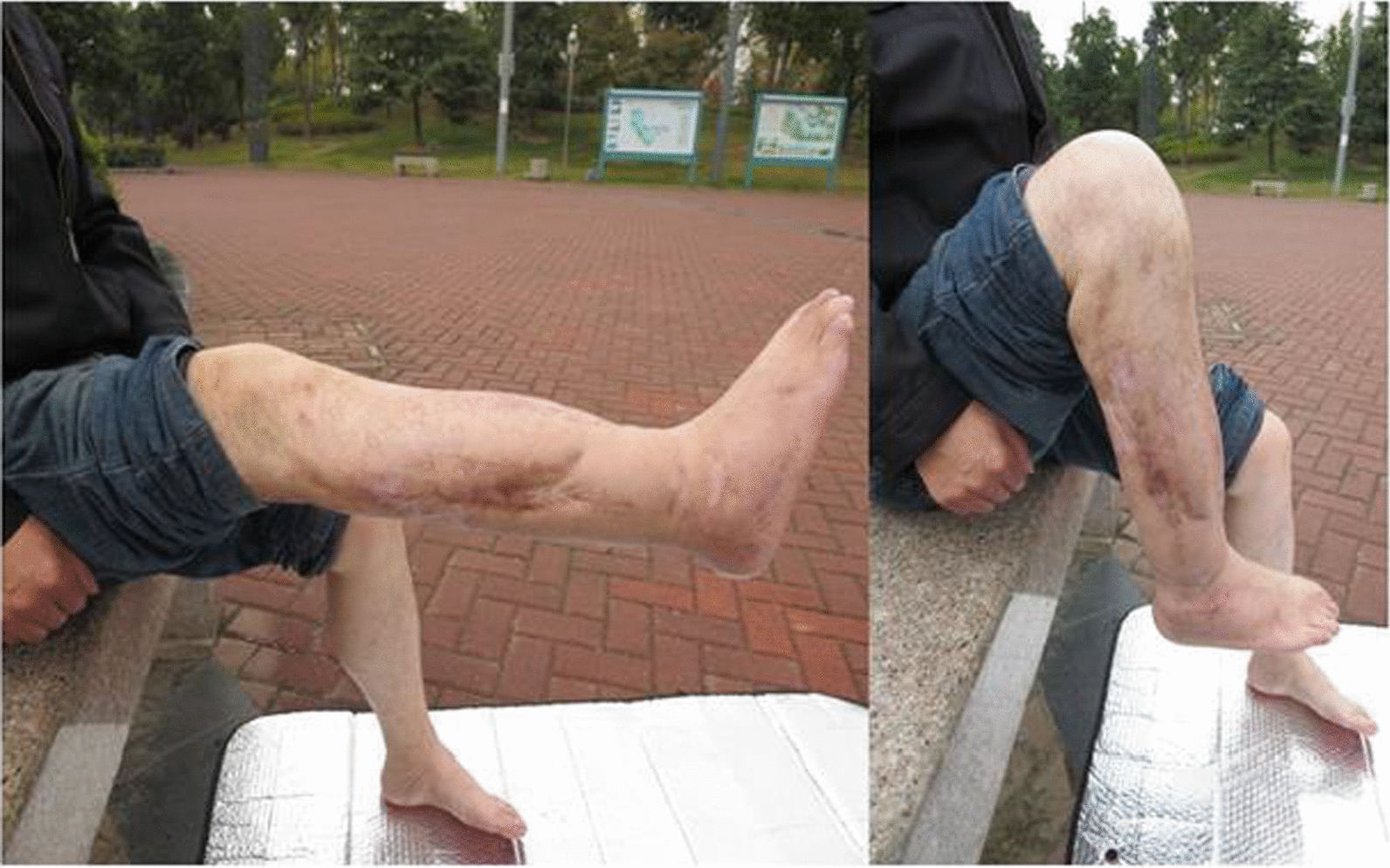
Patients shown in Fig. 4 had equal limb length at 18 months postoperatively. The functions of the knee and ankle were good, with an Enneking score of 30

Figure 8 showed the recovery of the lower leg of the patient who had arterial injuries in Fig. 4. After a year and a half of recovery, the patient's leg regained normal physiological function.

## Discussion

Early fracture stabilization has many advantages: the procedure facilitates patient mobility, improves pulmonary toilet, decreases pain and thus the need for narcotics, decreases inflammatory mediator response and thromboembolic phenomena [18]. Using external fixation is less invasive, can achieve adequate stability, and provides good wound management access without compromising stability [4].

Our study aimed to investigate the limb salvage outcome, functional results of these limb-threatening injuries through external fixation treatment, and to discuss the case of patients’ functional recovery after external fixation. In our study, we revealed that the use of external fixation had a high success rate of salvage, a low incidence rate of complications. Our results also showed a decrease in pain and improved quality of life and function, and these changes remained stable at follow-up.

Treatments for surgical stabilization of lower extremity fractures included plate fixation, intramedullary nailing with or without reaming, and external fixation. The traditional static immobilization using a plate and screw system carried a high fixation failure rate [19]. And compared with external fixation, the former treatments entailed greater blood loss and required increased operative time [18]. Today, lower extremity fracture patients with vascular injuries are treated mainly by plate fixation and intramedullary nailing (with or without reaming). So far, no studies have investigated the outcome of using external fixation treating lower limb fracture patients with vascular injuries. Similarly, these patients' functional recovery (follow-up) has not been investigated.

The selection of either anastomosis or autologous vein graft in lower extremity fracture patients with vascular injuries has been studied extensively. Traditionally, in free flap cover of lower limb injuries, performing anastomoses proximal to the zone of injury was recommended [20]. And autologous vein graft was used when anastomosis cannot be performed. Our study supposed that end-to-end anastomosis under the microscope is highly recommended in patients with vascular injuries. If interposition grafts are required, autologous vein graft is preferable to synthetic material, because a reversed saphenous vein graft from the contralateral limb has clearly superior patency rates, also can avoid foreign body in vivo [21–23]. Also, it is necessary to use computed tomography angiography to verify the area of vascular injuries [24]. But we should pay attention to the timing of the angiography. Asterios and his colleagues suggested that intraoperative arteriography should be used in patients with vascular injuries instead of preoperative diagnostic arteriography [25]. We agree with their view that intraoperative arteriography can be performed easily and quickly and is also noninvasive and requires less radiation [26]. This approach can be time-saving, which is vital in saving patients’ limbs and their lives.

Except for the selection for arterial repair and the use of angiography, re-vascularization is another significant factor related to long-term functional outcomes. Previous study [27] introduced the concept of the 6-h rule for re-vascularization, and most authors used 6 h as the definition of early intervention. However, skeletal muscle and nerve are, in fact, even more sensitive to ischaemia [28, 29]. Glass et al [30] in a Kaplan–Meier survival curve analysis, demonstrated that limb salvage begins to fall almost immediately the time when any further delay results in a rapid decline in survival, which begins at about 3–4 h. So, the time of re-vascularization should be as short as possible to minimize ischemia time and re-perfusion time, thus preventing potential necrotic changes and ischemia reperfusion injury, which is the key to limb salvage.

We should pay more attention to complications in using external fixators. Pin tract infection is one of the most common complications of external fixation. Occurring in 10.3% of our patients is comparable to those of 9.4% to 30% reported by other studies [31, 32]. Infection varies from minor inflammation remedied by local wound care,to superficial infection requiring antibiotics, local wound care, and occasional pin removal; to osteomyelitis requiring sequestrectomy. Higher rates of pin tract infection are seen when the pins are placed through large volumes of soft tissue (for example, thigh) [4]. A potential explanation is that pins placed within the zone of injury allowed bacteria to invade potential space created by soft-tissue disruption. So external fixator pins should be applied outside the zone of injury to span the zone of injury to minimize soft-tissue insult.

Pin-bone interface loosening or failure mainly with bone resorption around needles, full weight-bearing too early, fracture gaps > 2 mm of unstable fracture, osteoporosis, etc. External fixation failure includes the pins and link rod crack and bending deformation. Repeating bending makes the metal fatigue, which is a major cause of external fixation failure [33].

Another two common complications of external fixation are bone nonunion and deep infection developed osteomyelitis. For the bone nonunion, our study showed that bone nonunion rate was 20% (16/80), though we used the typical approach after removal of the external fixator, which included curettage, debridement, and irrigation of the pin sites with adjunctive antibiotic coverage [34]. One explanation could be that the original restoration was not satisfied, fracture gaps too large, and severe soft tissue injury. Cross and Swiontkowski [35] demonstrated as high as 13% bone nonunion rate using the external fixator, which was related to improper surgical technique, inaccurate fracture reduction, the initial severe trauma and the lack of elastic fixation changing in time. Menon [36] demonstrated the early period static fixation, middle and later period elastic fixation could be beneficial to the bone union. So, the ideal fracture reduction should be performed as well as possible before placing pins, instead of excessive dependence on external fixator adjusting.

For the deep infection, it is among the most important problems of open fractures, and the wound environment is very suitable for the spread of bacteria and this rate can reach 52% in Gustilo Type 3B injuries [37]. In our study, a low incidence of deep infection was observed (3.4%). We agree with Bilir et al. and our study indicated that the place of antibiotherapy is essential in the accurate treatment of these patients [37].

Except for these complications, we pay attention to another severe complication: amputation. So far, there have been several studies investigating whether MESS or other scales can be used to predict the outcome of lower limb fracture patients. Lin et al. agreed with the idea that the = threshold for immediate amputation can be raised from MESS = 7 to MESS = 9 [38]. Whether Zhou supposed that patients with MESS > or = 7 are more likely to undergo amputate their limbs [39]. However, Alexandra proposed that considering the significant advances in reconstructive techniques, decision-making in patients with a MESS of 7 or greater should be reevaluated for everyday clinical use [40]. Except for MESS, Gupta and his colleagues considered that Ganga hospital score (GHS) has an improved ability to determine amputation in IIIB open tibia fractures [41]. More interestingly, Andrew and his colleagues supposed that there was no significant difference between MESS values of amputees and those treated with limb salvage [42]. We supposed that high MESS score is related to the amputation, for high MESS score represents severe injuries to a certain extent. As for Gupta’s viewpoint, we held the view that a 3–2–1 modification of the Gustilo type IIIB classification to incorporate degree of arterial injury should be proposed [43].

Functional recovery is also very important. In order to improve the long-term outcome for our patients, we designed several approaches. Firstly, we should use the external fixation properly. Our study suggests that we should choose the proper fixator to improve patients’ prognosis rather than use external fixator without considering patients’ situation. And we agree with the idea that we should pay attention to the dynamization of fracture fixation to improve the fracture healing process [44]. Secondly, the combination of external fixation and other fixations may be much more beneficial in patients. Zhao demonstrates that combined fixation is an effective and safe alternative for the management of open tibial diaphyseal fractures compared with external fixation [45]. In clinical application, we may choose the proper fixation method depending on types of fracture, ages of the patients, the MESS or other scores, etc. Thirdly, we should pay more attention to patients with high MESS scores and we should try to propose personalized treatment protocol. Lastly, emotional factors play an important role in influencing patients’ prognosis. Previous studies showed that posttraumatic stress disorder, depression, and psychological disorders are common complications observed in patients with these devastating injuries [46, 47]. Lowering patients’ emotional pressure may be beneficial to patients’ prognosis.

Our study has some limitations. First, we didn’t conduct a subgroup analysis subdivided by soft tissue injury or type of vascular injury. Thus, we may not able to propose a standard or a personalized treatment protocol. Second, a multivariate analysis was not performed in the entire patient population. As a result, we couldn’t identify whether multiple factors affect the success rate of surgery, the rate of complications, the outcome of our patients, etc. That is what we’re going to do next.

## Conclusion

The external fixator has the advantages of reliable immobilization, can lower the possibility of fracture or dislocation in a short time, which creates a suitable environment for vascular repair and shortens limb ischemia time. By correctly judging the condition of limb ischemia, mastering the operation indications reasonably, and preventing complications, the use of external fixators to treat lower leg arterial injury can obtain better clinical effects. Here, we provide a relatively standardized process by which clinicians can correctly classify the fracture in order to apply appropriate external fixation methods to improve patients’ prognosis.

## Data Availability

The datasets used or analyzed to support the findings of this study are available from the corresponding author on reasonable request.
